# A Mobile App Adopting an Identity Focus to Promote Physical Activity (MoveDaily): Iterative Design Study

**DOI:** 10.2196/16720

**Published:** 2020-06-15

**Authors:** Floris Hooglugt, Geke D S Ludden

**Affiliations:** 1 Department of Design, Production and Management University of Twente Enschede Netherlands

**Keywords:** research through design, physical activity, habits, identity, behavior change, mHealth, design

## Abstract

**Background:**

Web-based and mobile interventions to influence physical activity behavior have had limited effects on sustained behavior change. One reason may be that the interventions aim to change largely habitual behavior. Following an identity-oriented approach could be a successful strategy to behavior change because people are committed to behave in line with their self-perception of identity.

**Objective:**

In this paper, we take a closer look at the role of motivation in long-term adherence to lifestyle interventions. The paper outlines a method for web-based or mobile intervention development that allows exploration of integrating behavior change theory into the design process. We will describe the development of a mobile app that allows people to be self-determined and to value and self-regulate physical activity by adopting an identity-oriented approach.

**Methods:**

This paper describes a Research through Design (RtD) process in which design activities are carried out as part of the knowledge-generating process. Two RtD phases were completed, followed by a conceptual design phase. In the first RtD phase, 8 participants used diary cards to study initial attitudes toward starting with small changes in physical activity. In the second RtD phase, 26 participants used a web-based app to study changes in physical activity. We used an adapted version of the Self-Report Habit Index (SRHI) to evaluate individuals’ perceptions of a particular behavior with respect to the three facets of a habit. The conceptual design phase consolidated the results from first two RtD phases into a design of a mobile app that combines an identity approach with gamification principles. The conceptual design was evaluated in a user-experience study with 4 participants.

**Results:**

In the first RtD phase, we found that interacting daily with diary cards and reflecting on physical activity patterns is a promising strategy but works better through a digital medium. In the second RtD phase, SHRI ratings from all participants generally increased each week. In the conceptual design phase, we found that the concept of the mobile app was positively evaluated by participants. However, participants mentioned that terms such as “identity” do not resonate with them and that scenarios could be simpler.

**Conclusions:**

This paper provides deeper insights into designing for electronic health (eHealth) interventions and services and suggests a new way that motivation can be shaped by the design of an intervention and adherence to physical activity. To the best of our knowledge, this was the first iterative design study in which the effects of adopting an identity approach to both motivation and physical activity were included and observed. Initial promising results were found for using a web-based intervention where habits and identification with the personal importance of a behavior were repetitively triggered.

## Introduction

### Background

As a response to the worldwide increase in so-called *lifestyle diseases* such as obesity and diabetes [1], efforts to raise people’s awareness of the importance of living a healthier life, as well as to motivate and support people to make lifestyle changes, have widened from traditional information campaigns to monitoring and coaching systems [2,3]. As a result of this, we have seen a multitude of web-based and mobile interventions as well as dedicated trackers, designed to support people in adopting a healthy lifestyle, reach the market [4,5]. Interventions are typically aimed at adopting a healthier diet or becoming more physically active and, in some cases, a combination of these two.

Although multiple studies [6,7] describe the effectiveness of web-based and mobile interventions on influencing physical activity behavior, others describe the uncertainty of their efficacy [8]. To date, it remains unclear which behavior change techniques lead to greater intervention success in increasing physical activity [7,9-14]. In addition, while a wide range of behavior change interventions exist that use similar techniques, studies have concluded that these generally lack the use of theoretical constructs [15-17]. It is worth noting that at the same time, we know that successful interventions are informed by grounded behavior change theory [18,19]. Furthermore, adherence to an intervention is essential for a positive effect on health to transpire [20], yet web-based and mobile interventions have not been as successful as typically traditional individual and or group treatment approaches for long-term adherence [21-23].

It seems difficult to single out one specific reason for the lack of success of interventions in achieving long-term behavior change. Several researchers have proposed that the affective experience of persuasive technologies is the key to their effectiveness [24,25]. In other words, a better design that is not only functionally effective but also desirable and engaging could improve acceptance of, and thereby adherence to, interventions [20]. Another reason for the lack of success may be that interventions try to target behaviors that are largely habitual in nature [23], often occurring outside the conscious awareness of people [26]. Many energy balance–related behaviors (eg, unhealthy eating) are performed habitually, with little forethought [27]. Interventions that focus on breaking these established habits will, therefore, face difficulties in the long term because people behave according to their habits even when motivated not to do so [28].

In this paper, we take a closer look at the role of habits and motivation in long-term adherence to lifestyle interventions. We will explore how the design of a mobile app can incorporate a focus on self-directed motivation. A self-determined form of motivation is regulation through identification. Over time, identified regulations can be fully adopted by a person as belonging to him or her [29]. Identification occurs when the person has identified with the personal importance of a behavior and has, thus, accepted its regulation as his or her own. An individual who always takes the stairs instead of the elevator because she or he sees it as relevant to her or his health, which she or he values as a life goal, has identified with the value of this physical activity [30]. Studies in relation to smoking behavior have shown that stronger smoker self-identity (ie, thinking of the self as a person who smokes) predicts fewer quitting attempts [31,32]. People are committed to behave in line with their self-perception of identity and, therefore, behavior change and identity change depend upon each other [33].

### Habits and Identity

**Figure 1:**
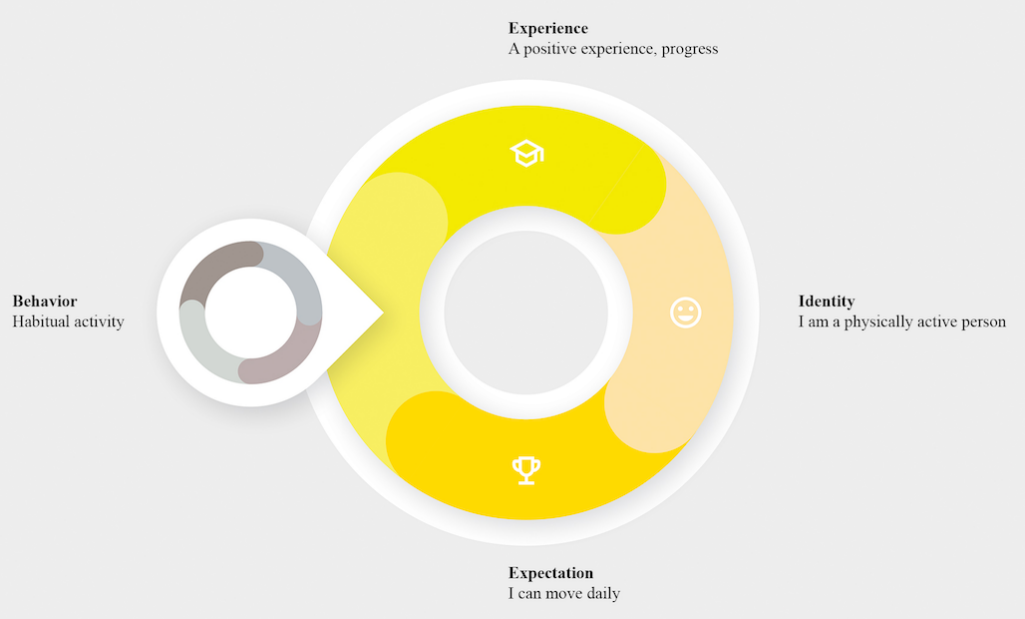
The relationship between the identity loop and the habit loop.

By experiencing positive feedback on daily habits, new beliefs about one’s identity can be formed. We visualized the relationship between habit forming and identity as two overlapping loops: the habit loop and the identity loop (see Figure 1). Any habit follows a closed loop of trigger, action, and reward [34,35]. The habit loop is part of the identity loop, showing how behavior that is performed consistently forms the starting point of changing one’s beliefs. Concurrently, the identity loop visualizes how beliefs can, in turn, influence behavior [33]. The identity loop has four phases (see Figure 1): behavior, experience, identity, and expectation. Following a performed activity (ie, the habit), an experience will cause a belief about one’s identity to be formed or reaffirmed. Out of that belief, an expectation about future behavior takes shape. A positive experience will allow for individuals to become more self-determined about the activity and for internalization to occur. Subsequently, the expectation of successfully performing the habit the next time becomes more realistic. If the behavior is repeated, it will lead back to a similar experience or result, which will reinforce one’s beliefs again. It is conceivable that, initially, these identity-focused habits will leverage extrinsic motivation via identification and progress over time to self-regulated integration.

By exploring the development of habits and a change in thinking about identity, we intend to find out how the design of a mobile app might support a sustainable increase in physical activity. The intervention focuses on creating habits through starting small, following the idea that if one’s ability is high (ie, by starting with a simple activity), motivation can be low, yet an individual will still be able to perform the behavior [36].

### Objectives

The primary aim of this paper is to outline a method for web-based or mobile intervention design and development that allows for integration of behavior change theory in the design process. We will describe the development of a mobile app that allows people to be self-determined and to value and self-regulate physical activity by adopting an *identity-oriented* approach. The design process and the iterative evaluation of this app called MoveDaily can serve as an example of a holistic approach to design for behavior change. In this way, this paper contributes to knowledge on the value of adopting an identity approach to behavior change interventions aimed at increasing physical activity. Furthermore, it describes an iterative and user-centered Research through Design (RtD) process [37-39], thereby adding to the body of knowledge on how designers can work with behavior change theory and strategies while designing for behavior change. The iterative evaluation and experimentation with the prototypes will provide knowledge about how features of the design influence people’s motivation and behavior. We will discuss both insights gained through the design activities and those gained through evaluation of the effects of the early prototypes. Together, they will contribute to insight on how people respond to an identity approach to behavior change. Our RtD process is comprised of three phases; we will discuss methods and results in the corresponding sections for each of the three phases and end with a general discussion of the insights and results obtained throughout the three phases.

## Methods

### Research Through Design

**Figure 2:**
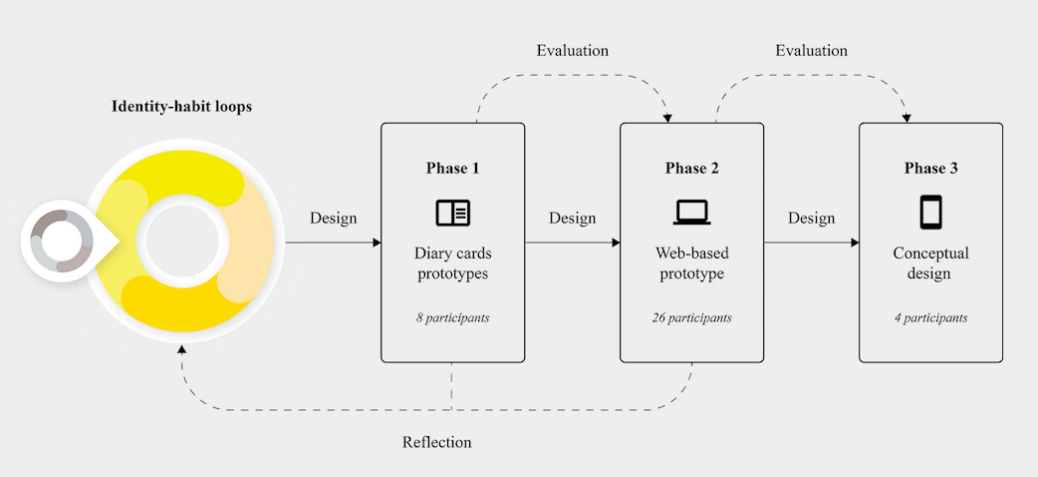
Flowchart of the Research through Design (RtD) process for the mobile app MoveDaily.

In an RtD process, design activities are carried out as part of the knowledge-generating process. They often include the development of early prototypes that could be mistaken for a *product* but that are specifically designed to generate knowledge. The process of translating the abstract concepts of identification into designed elements or features of an app is considered an important part of the work [39]. Our RtD process consisted of two main phases that were followed by a conceptual design phase (see Figure 2). The fidelity of the created prototypes increased with each sequential phase. During the first phase, participants’ initial attitudes toward a paper intervention (ie, diary cards paper prototypes), in which they started with small physical activities, were observed. During the second phase, participants’ attitudes toward a web-based intervention (ie, web-based prototype) were studied, as well as participants’ needs and desires for longer-term use. The following design phase described the conceptual design of the mobile app MoveDaily.

### Phase 1: Diary Cards Prototypes

#### Overview

The aim of this phase was to explore how to perform a first translation from the framework in Figure 1 to a design and to obtain insight into initial attitudes of participants about increasing their physical activity.

**Figure 3:**
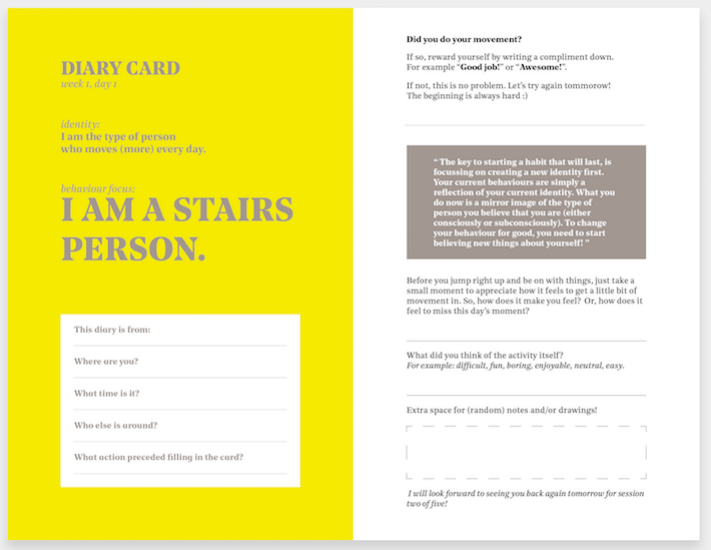
Example of the diary cards used in week 1.

In this first RtD phase, we studied initial attitudes toward starting small and whether this attitude could change over time. During this phase, we evaluated four intervention types over a 4-week period. The interventions’ features changed every week and were designed with the aim to learn about participants’ attitudes and opinions. The first three interventions were designed as diary cards that changed every week and that were given to the participants daily as reminders of being physically active. Every week the intervention was adjusted to better fit the needs of the participants. The diaries were designed in a way that allowed for a simple and pleasurable interaction, while also being easily adjustable for each week’s variation; see Figure 3 for an example of the diary cards. In the first week, diary cards were aimed at achieving a mindset change in regard to starting small. In the second week, the cards were aimed at supporting participants to determine for themselves at what rate to increase the amount of activity of their routine. In the third week, participants were expected to start enjoying their small activities. In the fourth week, a digital form of the intervention was introduced to evaluate whether using the intervention via a digital medium was preferred over paper.

#### Participants

A small number of participants was involved because the aim of this phase was to explore what would be the best format of the intervention to integrate the identity-habit loops. Recruiting participants was done internally within a company through announcements via the digital medium Slack. Participants included 8 native Dutch-speaking employees: 5 women (63%) and 3 men (38%) in their 30s and 40s.

#### Protocol

Before starting the trial, participants chose between three different identities and were asked to select the one that they would like to adopt. The options were as follows: “I am a person who walks” (*walking*), “I am a strong person” (*strength*), and “I am a stairs person” (*taking the stairs*).

Participants reported to the researcher about their physical activity daily. Additionally, short interviews were held weekly to understand each participant’s attitudes toward that week’s diary cards. The discussion topics were partly based on what participants reported on their diary cards, but questions were mostly asked about how easy it was for each participant to perform the physical activity (ie, automaticity for an indication of habit strength) and if they wanted to increase the difficulty of the physical activity. When a participant decided to discontinue the trial, an exit interview would take place. This interview was mainly aimed at gathering feedback from the participants on how to improve the intervention.

### Phase 2: Web-Based Prototype

#### Overview

**Figure 4:**
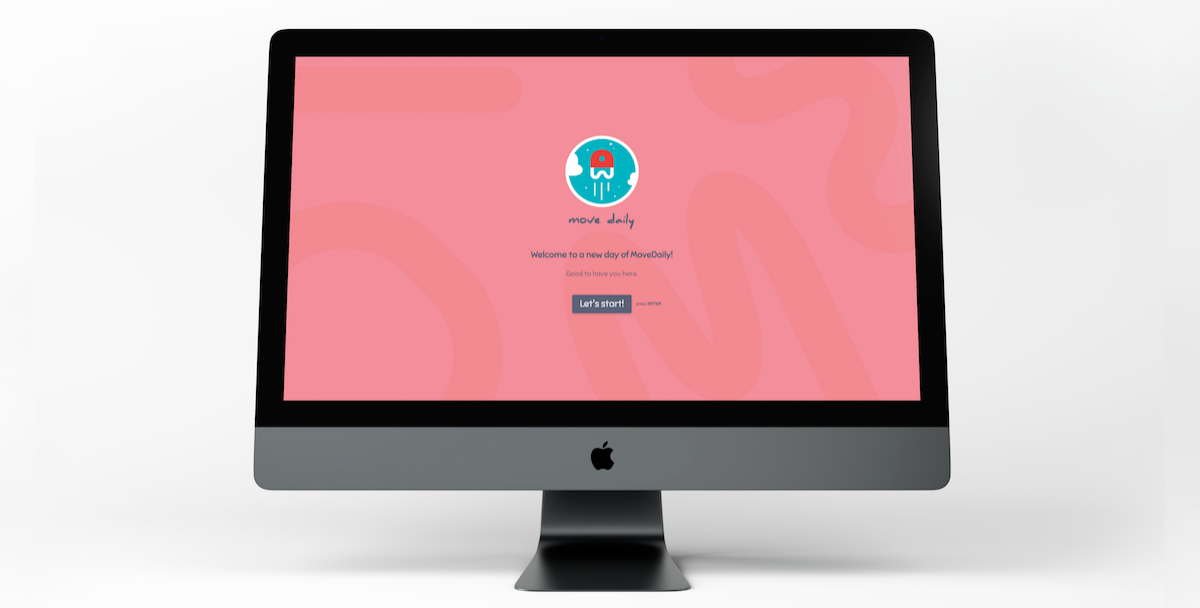
Screenshot of the web-based prototype for MoveDaily.

In this second RtD phase, we studied participants’ reactions and behavior using a simple web-based intervention. Lessons learned in the first RtD phase were incorporated into a digital prototype in the online survey software Typeform. The main feature of the web-based prototype was to present users with a daily reminder of the identity they selected (see Multimedia Appendix 1). The intervention was presented to participants as an actual service named MoveDaily. It has a bold and playful identity, with fitting color schemes (see Figure 4). Both qualitative and quantitative data were collected to study the participants’ views and attitudes as well as their self-reported behavior. Specifically, we used both a questionnaire and follow-up interviews. Following Schmidt and Retelsdorf [40] and Verplanken et al [41], an adapted Self-Report Habit Index (SRHI) model [42] was used in the questionnaire to report on habits regarding physical activity (SRHI-P) (see Multimedia Appendix 2). Responses were made on 7-point Likert scale ranging from 1 (*strongly disagree*) to 7 (*strongly agree*).

Participants who continued to use the intervention were expected to strengthen their habits over time, meaning the longer the participants adhered, the more consistent their physical activities would be. First, a high SRHI-P score was expected to correlate with a high score on physical activity frequency. Second, a high SRHI-P score was also expected to be associated with more positive remarks by the participants in the interviews and questionnaires regarding the web-based intervention. Third, positive correlations between whether participants recognized feeling free of any pressure or performance and the amount of physical activity performed were expected. Fourth, positive correlations between early dropouts and negative remarks about the intervention (eg, *not fun*) were expected. Fifth, a stronger physical activity habit should not only lead to more physical activity but also to a stronger personal image as someone who is comfortable with physical activity. Therefore, positive correlations with physical activity achievement and taking up extra movement were expected.

#### Participants

There was a total of 26 participants, including 22 native Dutch-speaking university students (85%), 1 Portuguese student (4%), 1 French student (4%), and 2 Dutch working people (8%); the sample was made up of 15 women (58%) and 11 men (42%), aged 17-63 years (mean 25 years, SD 4). Participants were recruited by making use of a convenience sample, in which the subjects were selected because of their convenient accessibility and proximity to the researcher, and snowball sampling, which made use of recruitment of participants by other participants already in the study (ie, the previous RtD phase). Psychology students were ineligible for the study to ensure questionnaire interpretation could not be biased by psychology training.

This study methodology does not impose direct sample size constraints; therefore, no cap was placed on the number of participants. A sample of 20 participants was deemed likely to capture a broad-enough range of viewpoints and problems and was, therefore, the goal. We were able to recruit 26 participants. The testing period was 10 weeks in length: 50 habit-forming days and 20 rest days.

#### Protocol

The online platform Typeform was used for the intervention and as the main data-gathering tool. Typeform is a web-based platform for collecting and sharing information, in a “conversational, human way.” It allows for the fast adjustability that was necessary, as the MoveDaily prototype needed to be adjusted daily in order to appear flexible and smart. Typeform was also capable of saving data input from participants. Email and WhatsApp were adopted as the main tools used for daily interaction with the users, while interviews were done through phone calls.

To start using the intervention, participants signed up by completing an 18-item questionnaire to gather demographics and to allow them to choose between three behaviors: *taking a walk*, *climbing the stairs*, and *doing exercises*. Each participant selected one behavior as the focus for the intervention. A short tutorial of the intervention followed, and a video explained how starting small can be promising.

Participants received daily messages via email at the time specific to their exercise time. Furthermore, participants reported about their activities daily through Typeform. Every week a random selection of 2-3 participants was interviewed about their experiences and the interview data were manually noted down. If participants decided to discontinue using the intervention, they were asked to fill out a dropout form. After participants filled out this form, a follow-up interview was held to confirm understanding of the reason for dropout.

### Phase 3: Conceptual Design

#### Overview

By consolidating the results from RtD phases 1 and 2, a conceptual design for the MoveDaily app was made. MoveDaily is an identity-oriented app supporting sustainable habits around physical activity. The app is shaped like a simple explorer game, leveraging gamification as a means to achieve initiation and retention of desired behaviors [43-45]. The conceptual design of MoveDaily was used in a usability and user-experience evaluation; the interactive prototype can be accessed online [46].

Before designing MoveDaily, we specified what the conclusions of the two RtD phases meant for the design of a mobile app. This led to the following five design guidelines:

1. Activate the user from the start by designing the habit during the first time of use.
2. Create a performance-free product, stimulate physical activity without introducing the notion of sport or exercise, and emphasize the benefits of starting small.
3. Increase understanding about habit forming. By doing so in a playful manner, people can start to understand how their healthy behavior can be strengthened and why we start with small activities.
4. Change users’ mindsets from goal oriented to identity oriented.
5. Design for daily interaction.

**Figure 5:**
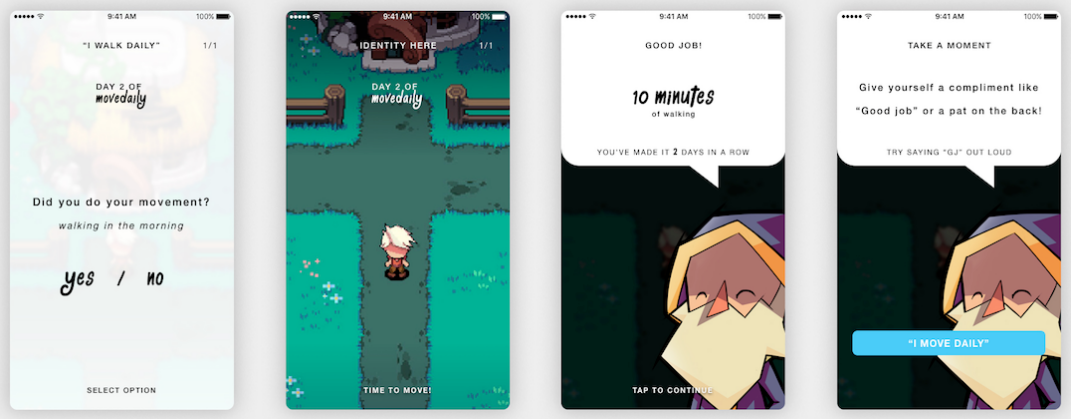
A reaffirmation example of the app MoveDaily. If an activity was performed successfully, the user is complimented and reminded of his or her daily steps forward.

**Figure 6:**
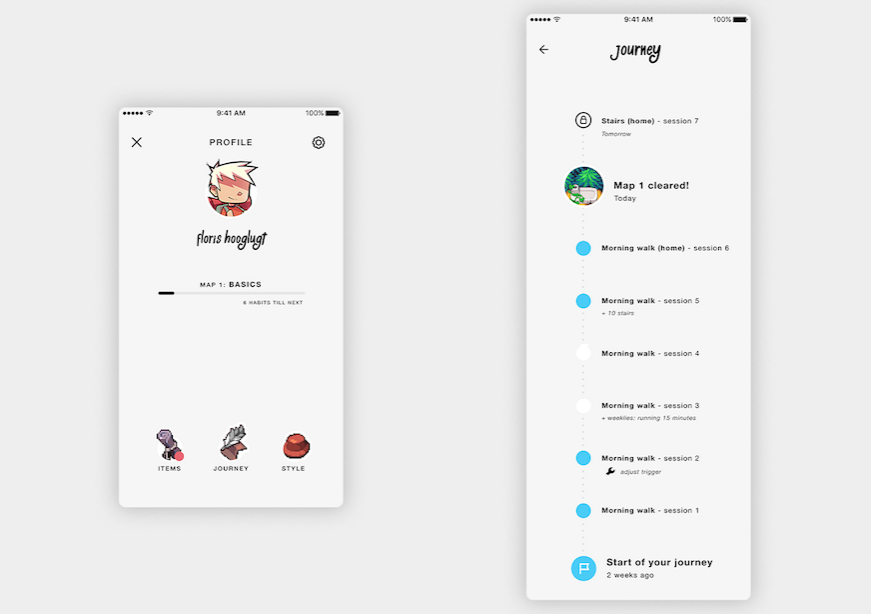
Two examples of the app MoveDaily as to how progress can be visualized: through a level indicator of the avatar (image on the left) and through a timeline showing all performed activities (image on the right).

In Figure 5, an example of a feature derived from the design guidelines is shown, where the user is prompted to reaffirm to themselves that they can be a certain kind of person. Due to this *self-reevaluation* after the activity, people assess how they think and feel about themselves with regard to the performed behavior and create a new self-image (eg, Prochaska et al [47] and Velicer et al [48]). In addition to the five design guidelines, knowledge on successful persuasive features informed the design [49]. An example of such a feature is a visual representation of a user’s progress for positive reinforcement (see Figure 6).

**Figure 7:**
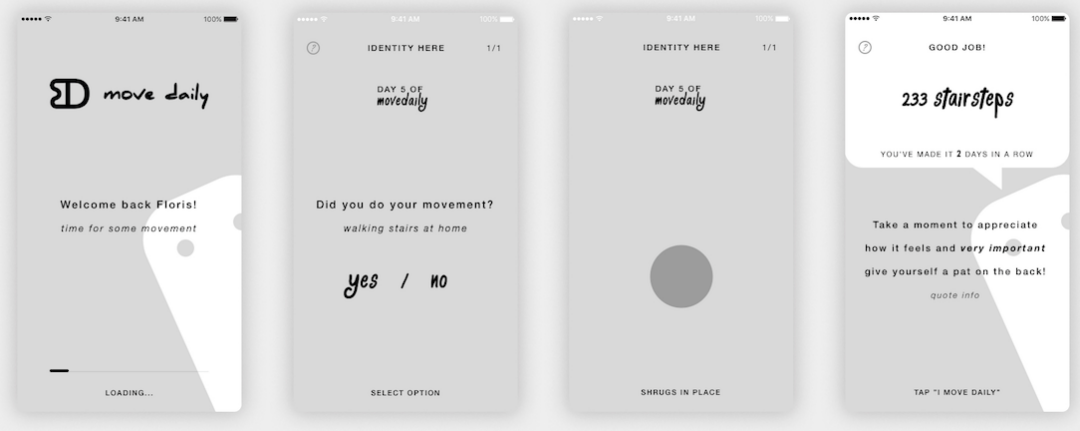
Selection of wireframes informing the final design of the mobile app MoveDaily.

To come to a high-fidelity conceptual design, we started by sketching variations of app structure, interactions, and layout on paper. Following this phase, wireframes were created in Sketch, a digital design toolkit (see Figure 7). Wireframes allow the designer to further experiment with ideas and concepts on a higher-fidelity level and allow for checking the interaction flow. Next, the wireframes were visually designed and an interactive prototype was created in the workflow tool InVision (InVisionApp Inc).

#### Participants

Participants were recruited by making use of convenience sampling. We recruited 4 participants, including 2 native Dutch-speaking university students (50%) and 2 Dutch working people; the sample was made up of 2 women (50%) and 2 men (50%), aged 22-31 years. Besides no prior experience with the project, no exclusion criteria were used for participant selection.

#### Protocol

The interactive prototype of MoveDaily was installed on an iPhone 6 device and the prototype was presented to participants during a single 45-minute session. After presenting a use scenario, participants were asked to interact with the prototype. Four scenarios were provided sequentially: (1) first-time use, (2) being successful in performing the daily habit, (3) being unsuccessful in performing the daily habit, and (4) personalizing the experience. After each scenario, questions were asked regarding the experience with the app. Following usability testing guidelines [50], the interviewer was reminded to keep neutrality in response to user comments and behavior and read the script to each user in the same way. A notetaker observed and kept track of user comments and behavior. We relied on the qualitative analysis of user feedback for this study.

## Results

### Overview

To explore how people respond to an identity approach to behavior change, we adopted an iterative design approach of three phases, increasing the fidelity in each sequential phase. In this section, we summarize the results of these three phases, specifically the self-reported data of the diary cards prototypes, self-reported feedback through the web-based prototype, the follow-up interviews, the conceptual design of the MoveDaily app, and the result of a usability test.

### Phase 1: Diary Cards Prototypes

A total of 8 participants took part in the diary cards study. Out of 8 participants, 2 (25%) dropped out within the first week. Out of the 6 remaining participants, 1 (17%) chose to leave the study at one point during the 10 weeks. A total of 4 participants from the original 8 (50%) opted for the walking program, while the other 4 (50%) opted for the exercise (ie, strength) program. No participant opted for the stairs program; most participants found this exercise to be too tiring. Transcripts of the interviews were made and these were analyzed by tallying the most common topics and themes.

From the self-reported data, we can observe that participants generally liked how they were conscious of their daily activity through the diary cards (see Table 1). While some described them as practical and simple, one participant also mentioned in one of the early interviews, “It feels like the cards attempt to put an application’s functionalities within paper. I would prefer digital reminders, now I have to be aware of the piece of paper.” The first prototype was not favored as it took too long to complete, nor was it experienced as fun. A total of 5 participants out of 8 (63%) struggled remarkably more than the others with remembering to fill in their diary cards. Cards that had a shorter completion time and features enhancing pleasure, such as rewarding yourself with a sticker, were evaluated more positively. While the digital medium used in the fourth week was limited in its possibilities, it did confirm the benefits of a digital medium versus a paper-based intervention. As expected, initial reactions of participants toward starting small were negative. Participants initially wanted to start with, for example, 40 squats. Surprisingly, all participants increased the difficulty of their exercise only by small margins weekly.

### Phase 2: Web-Based Prototype

In phase 2, 26 participants were included. We observed diverse reactions to the web-based intervention. Out of 26 participants, 5 (19%) dropped out within the first week. A total of 10 of the 21 remaining participants (48%) decided to leave at a later time during the study, citing various reasons. Furthermore, while some participants successfully performed and maintained their activity from the beginning, others were unable to consistently commit to the intervention. We included data received from participants who dropped out before the end of the study in our analyses.

To study the participants’ responses from the self-report and interview data, the most common topics and themes were tallied (see Table 1). Results from analysis of this data indicated that the MoveDaily concept was received positively by the participants. While not all participants seemed to understand the concept fully, almost all would mention some key aspects of MoveDaily, such as “habit forming,” “learning a new behavior,” “free of pressure,” “starting small,” or “personal.” It was difficult for the participants to specifically indicate how these concepts became apparent to them, but answers such as “the message on the bottom of the mail,” “the introduction movie,” or “the whole thing” were frequently given.

To evaluate whether people rated the strength of their habits higher over time, a linear regression analysis was calculated to predict SHRI-P based on time (ie, week). A significant regression equation was found (*F*_1,7_=9.644, *P*=.02) with an R^2^ of 0.58. A more detailed inspection of the data did reveal that there were large differences in the way participants assigned scores to rate the strength of their habits. The SRHI-P scores from the first week, for example, ranged from 1.5 to 5.5 (mean 3.2, SD 1.12). Participants explained that they rated the strength of their habits as lower when they had not performed their physical activity that week. Nevertheless, the rising trend supports the idea that habits were successfully formed.

The weekly mean for the identity scores indicate whether participants think the exercise is becoming more “typically them.” Again, a linear regression analysis was calculated to predict identity based on time (ie, week). A significant regression equation was found (*F*_1,7_=16.938, *P*=.003) with an R^2^ of 0.74. This result indicates that participants successfully formed new beliefs about themselves.

Participants mentioned that they struggled with the concept of starting with small activities. Participants who successfully adhered for a longer period of time did change their attitudes toward MoveDaily over time; participants developed new experiences with MoveDaily. For example, 7 participants out of 26 (27%) who indicated during the initial interviews that they liked the concept, yet missed the competitive aspect, eventually dropped out. However, 6 other participants out of 26 (23%), who similarly indicated missing the competitive aspect, finished the 10-week period due to their interest in developing automaticity in physical activity. When continuing, these participants developed similar attitudes as those who were positive about the concept from the start.

The interview data also indicated that participants became more aware of how they could integrate small activities into their daily lives. For example, whereas one of the participants used to not think about taking the stairs, elevator, or escalator, often resulting in not taking the stairs, she now very consciously took the stairs. This awareness might come from the focus on a certain identity, that is “I am a stairs person.” In other cases, participants seemed to become more aware of their health in general, similar to what occurred with the diary cards. One participant explained about his recent contact with a physiotherapist, another participant became a bit more aware of his diet, and sometimes participants even picked up sports (eg, tennis) during the period of the study.

### Phase 3: Conceptual Design

Data gathered during the user evaluation of the conceptual design of MoveDaily indicated a general positive reaction to the app. During observation, participants showed the ability to go through the different interaction scenarios and complete the required tasks with relative ease. While interacting with the prototype, all 4 participants (100%) indicated an interest in learning more about the habit-forming aspect of the product and appreciated the alternative approach of starting with small activities (see Table 1). The two most-pressed issues for improvement did not directly relate to the introduced identity approach itself. Participants indicated that they would recommend to (1) improve the user interface of the home page and (2) improve the copywriting within the app. Overall, participants seemed pleased with the usability of the high-fidelity prototype and with the identity-oriented concept.

**Table 1:** List of core themes and relative example quotes derived from qualitative analysis of the three phases.

Phases, theme groups, and themes			Example quotes
**Phase 1**			
	**Habit forming**		
		Starting attitude toward starting small	“Four squats mean absolutely nothing!”
		Conscious of the daily activity	“Seeing the value in starting small and how this can be built up, is quite motivating.”
		Awareness of habit construct	“By changing my routine, I was capable of doing my exercise successfully more often. Before I had struggles with time and clients dropping by.”
	**Identification**		
		N/A^a^	N/A
	**Other**		
		Importance of a fun factor	“I do not really have an opinion about the stickers, but it is for sure more fun than simply writing.” “It feels like the cards attempt to put an application’s functionalities within paper. I would prefer digital reminders, now I have to be aware of the piece of paper.”
**Phase 2**			
	**Habit forming**		
		Starting attitude toward starting small	“Sometimes I forgot whether or not I did my habit. If it would be more difficult, I would feel like it has more effect.”
		Ease of consistency	“I think I often want to move but am unable to find the motivation. So in this sense, the concept speaks to me.” “By doing my habit every day, I think I am strengthening the value of the thought ‘Yes, I did it again today. I am goood!’”
		Feeling free of any pressure or performance	“It is nice to do something daily for myself.” “It is also good for the average person; no gym talk like ‘You are a loser if you do not look like me.’ It guides you while providing enough freedom for you to create your own habit.”
	**Identification**		
		Strong self-identity	“I have not really started to move more, but I have become more aware of the things I am doing. Like taking the stairs at the station is completely engrained in my behavior. Even when I am carrying a heavy bag during vacation, I still do not want to give up on taking the stairs.”
		Adoption of a behavior	“I currently do not just take the stairs at work, I also take them at the station or wherever I am.”
		Support in rewarding	“I fill in the form quite unconsciously. I am not sure if I really need it anymore, but I always do like the reminder to give myself a small compliment.”
**Phase 3**			
	**Habit forming**		
		Interest in learning	“I would be interested in more habit-forming tips, for example, how to create better triggers.”
		Feeling free of any pressure or performance	“I appreciated the in-app feedback and how it explained that even when I failed, the behavior can be reshaped to fit me better.”
	**Identification**		
		Difficulties with technical phrasing	“‘Developing a new identity’ sounds a bit heavy to me.”
	**Other**		
		Engagement with gamification	“I like how you explore the world further, the more successful movements you make.”

^a^N/A: not applicable.

## Discussion

### Principal Findings

The first RtD phase (ie, diary cards) created a better understanding on how to translate the combination of the habit loop with the identity loop into a fitting and valuable intervention design. It also resulted in a confirmation that participants would prefer, and maybe even place more trust in, a digital intervention. In the second RtD phase, we found that high habit scores are indeed, as hypothesized, indicative of physical activity frequency. Similarly to forming habits in general, frequency is, thus, also important for forming strong identity-oriented habits. The third phase of this study, the conceptual design phase, showcases how an app that makes people more identity oriented could materialize. It is offered in the format of a functional game supporting people to become more physically active.

Taken together, the three RtD phases effectively show how theory on behavior change can be implemented in design by following an iterative approach. The presented RtD approach intends to show designers how the complex situation surrounding adherence to physical activity can be approached, how it can be framed and reframed, and how prototypes that address it can be iteratively developed. In this manner, the design approach allows for designers to learn what a design interpretation of a theoretical model could look like and what responses of future users might be. Thereon, the design could more easily be improved by a future design (ie, iteration).

This study followed an explorative and iterative approach. The number of participants was rather low and we mostly collected data based on self-report. Moreover, our participants were young and we did not measure or ask our participants about their fitness levels or amounts of daily or weekly exercise before participation. While this will have influenced the accuracy of the data on physical activity and limits extrapolation to a general population, our data provided rich insights about participants’ attitudes toward using the intervention and changing their behavior. In order to evaluate the long-term efficacy and influence of the inward orientation (ie, identity), a longitudinal study should be conducted that includes a more heterogeneous population and that collects actual log data from the mobile app and combines this with self-reports. Baretta et al [51] showed that such a longitudinal study can provide rich insights into how people experience certain app features and what they think is most beneficial to them over the longer term. Future research may also be aimed at determining what small exercises are most effective in changing people’s beliefs about themselves.

### Conclusions

It is clear that design can influence human behavior, yet the understanding of designing for behavior change is still fragmented [52]. This paper provides deeper insights into designing for electronic health (eHealth) interventions and services and suggests a new way to shape motivation through the design of an intervention and adherence to physical activity. It introduces a new model to address the issue of behavior change in relation to physical activity and demonstrates how this model can be used in an iterative design approach that focuses on the development of a mobile health (mHealth) app that supports being more physically active. In this way, the described process serves as an example of a holistic approach to design for behavior change.

To the best of our knowledge, this is the first iterative design study in which the effects of adopting an identity approach to both motivation and physical activity were included and observed. Through the presented approach, which combines the habit loop with the identity loop, we presented initial promising results toward understanding how sustainable behavior change can be achieved. Habits serve as an important base for behavior change by having someone identify with the personal importance of a behavior repetitively. Furthermore, we demonstrated how such a theoretical idea can be explored in a three-step iterative RtD approach.

## Appendix

Multimedia Appendix 1 Example of a daily interaction with the web-based prototype.

Multimedia Appendix 2 Self-Report Habit Index (SRHI) questionnaire.

## Abbreviations

eHealth: electronic health
mHealth: mobile health
RtD: Research through Design
SRHI: Self-Report Habit Index
SRHI-P: Self-Report Habit Index-physical activity

## References

[1] Lee I, Shiroma EJ, Lobelo F, Puska P, Blair SN, Katzmarzyk PT, Lancet Physical Activity Series Working Group (2012). Effect of physical inactivity on major non-communicable diseases worldwide: An analysis of burden of disease and life expectancy. Lancet.

[2] de Vries H, van't Riet J, Spigt M, Metsemakers J, van den Akker M, Vermunt JK, Kremers S (2008). Clusters of lifestyle behaviors: Results from the Dutch SMILE study. Prev Med.

[3] Prochaska JJ, Nigg CR, Spring B, Velicer WF, Prochaska JO (2010). The benefits and challenges of multiple health behavior change in research and in practice. Prev Med.

[4] Consolvo S, Everitt K, Smith I, Landay JA (2006). Design requirements for technologies that encourage physical activity. Proceedings of the SIGCHI Conference on Human Factors in Computing Systems (CHI '06).

[5] Consolvo S, Klasnja P, McDonald DW, Landay JA (2009). Goal-setting considerations for persuasive technologies that encourage physical activity. Proceedings of the 4th International Conference on Persuasive Technology (PERSUASIVE 2009).

[6] Hurling R, Catt M, De Boni M, Fairley B, Hurst T, Murray P, Richardson A, Sodhi JS (2007). Using internet and mobile phone technology to deliver an automated physical activity program: Randomized controlled trial. J Med Internet Res.

[7] Fanning J, Mullen SP, McAuley E (2012). Increasing physical activity with mobile devices: A meta-analysis. J Med Internet Res.

[8] Marcolino MS, Oliveira JA, D'Agostino M, Ribeiro AL, Alkmim MB, Novillo-Ortiz D (2018). The impact of mHealth interventions: Systematic review of systematic reviews. JMIR Mhealth Uhealth.

[9] Direito A, Carraça E, Rawstorn J, Whittaker R, Maddison R (2017). mHealth technologies to influence physical activity and sedentary behaviors: Behavior change techniques, systematic review and meta-analysis of randomized controlled trials. Ann Behav Med.

[10] Schoeppe S, Alley S, Van Lippevelde W, Bray NA, Williams SL, Duncan MJ, Vandelanotte C (2016). Efficacy of interventions that use apps to improve diet, physical activity and sedentary behaviour: A systematic review. Int J Behav Nutr Phys Act.

[11] Bort-Roig J, Gilson ND, Puig-Ribera A, Contreras RS, Trost SG (2014). Measuring and influencing physical activity with smartphone technology: A systematic review. Sports Med.

[12] Monroe CM, Thompson DL, Bassett DR, Fitzhugh EC, Raynor HA (2015). Usability of mobile phones in physical activity–related research: A systematic review. Am J Health Educ.

[13] Zhao J, Freeman B, Li M (2016). Can mobile phone apps influence people's health behavior change? An evidence review. J Med Internet Res.

[14] Stuckey M, Carter S, Knight E (2017). The role of smartphones in encouraging physical activity in adults. Int J Gen Med.

[15] Cowan LT, Van Wagenen SA, Brown BA, Hedin RJ, Seino-Stephan Y, Hall PC, West JH (2013). Apps of steel: Are exercise apps providing consumers with realistic expectations?: A content analysis of exercise apps for presence of behavior change theory. Health Educ Behav.

[16] West JH, Hall PC, Hanson CL, Barnes MD, Giraud-Carrier C, Barrett J (2012). There's an app for that: Content analysis of paid health and fitness apps. J Med Internet Res.

[17] Breton ER, Fuemmeler BF, Abroms LC (2011). Weight loss-there is an app for that! But does it adhere to evidence-informed practices?. Transl Behav Med.

[18] Murtagh EM, Nichols L, Mohammed MA, Holder R, Nevill AM, Murphy MH (2015). The effect of walking on risk factors for cardiovascular disease: An updated systematic review and meta-analysis of randomised control trials. Prev Med.

[19] Webb TL, Joseph J, Yardley L, Michie S (2010). Using the internet to promote health behavior change: A systematic review and meta-analysis of the impact of theoretical basis, use of behavior change techniques, and mode of delivery on efficacy. J Med Internet Res.

[20] Kelders SM, Kok RN, Ossebaard HC, Van Gemert-Pijnen JE (2012). Persuasive system design does matter: A systematic review of adherence to web-based interventions. J Med Internet Res.

[21] Krukowski RA, Harvey-Berino J, Ashikaga T, Thomas CS, Micco N (2008). Internet-based weight control: The relationship between web features and weight loss. Telemed J E Health.

[22] Bennett GG, Glasgow RE (2009). The delivery of public health interventions via the internet: Actualizing their potential. Annu Rev Public Health.

[23] Hermsen S, Frost J, Renes RJ, Kerkhof P (2016). Using feedback through digital technology to disrupt and change habitual behavior: A critical review of current literature. Comput Human Behav.

[24] IJsselsteijn W, de Kort Y, Midden C, Eggen B, van den Hoven E (2006). Persuasive technology for human well-being: Setting the scene. Proceedings of the International Conference on Persuasive Technology (PERSUASIVE 2006).

[25] Tromp N, Hekkert P, Verbeek P (2011). Design for socially responsible behavior: A classification of influence based on intended user experience. Des Issues.

[26] Hollands GJ, Marteau TM, Fletcher PC (2016). Non-conscious processes in changing health-related behaviour: A conceptual analysis and framework. Health Psychol Rev.

[27] Ouellette JA, Wood W (1998). Habit and intention in everyday life: The multiple processes by which past behavior predicts future behavior. Psychol Bull.

[28] Gardner B, de Bruijn G, Lally P (2011). A systematic review and meta-analysis of applications of the Self-Report Habit Index to nutrition and physical activity behaviours. Ann Behav Med.

[29] Ryan RM, Deci EL (2000). Intrinsic and extrinsic motivations: Classic definitions and new directions. Contemp Educ Psychol.

[30] Deci EL, Ryan RM (1985). Conceptualizations of intrinsic motivation and self-determination. Intrinsic Motivation and Self-Determination in Human Behavior.

[31] Høie M, Moan IS, Rise J (2010). An extended version of the theory of planned behaviour: Prediction of intentions to quit smoking using past behaviour as moderator. Addict Res Theory.

[32] Moan IS, Rise J (2005). Quitting smoking: Applying an extended version of the theory of planned behavior to predict intention and behavior. J Appl Biobehav Res.

[33] Kearney M, O'Sullivan J (2003). Identity shifts as turning points in health behavior change. West J Nurs Res.

[34] Duhigg C (2012). The Power of Habit: Why We Do What We Do in Life and Business.

[35] Eyal N (2014). Hooked: How to Build Habit-Forming Products.

[36] Fogg BJ Fogg Behavior Model.

[37] Stappers PJ, Giaccardi E (2017). The Encyclopedia of Human-Computer Interaction. 2nd edition.

[38] Zimmerman J, Forlizzi J, Evenson S (2007). Research through design as a method for interaction design research in HCI. Proceedings of the SIGCHI Conference on Human Factors in Computing Systems.

[39] Gaver W (2012). What should we expect from research through design?. Proceedings of the SIGCHI Conference on Human Factors in Computing Systems.

[40] Schmidt FT, Retelsdorf J (2016). A new measure of reading habit: Going beyond behavioral frequency. Front Psychol.

[41] Verplanken B, Myrbakk V, Vemund RE, Betsch T, Haberstroh S (2005). The measurement of habit. The Routines of Decision Making.

[42] Verplanken B, Orbell S (2003). Reflections on past behavior: A self-report index of habit strength. J Appl Soc Psychol.

[43] Deterding S, Dixon D, Khaled R, Nacke L (2011). From game design elements to gamefulness: Defining gamification. Proceedings of the 15th International Academic MindTrek Conference: Envisioning Future Media Environments.

[44] Fankhauser D (2013). Mashable.

[45] King D, Greaves F, Exeter C, Darzi A (2013). 'Gamification': Influencing health behaviours with games. J R Soc Med.

[46] InVision.

[47] Prochaska JO, DiClemente CC, Norcross JC (1992). In search of how people change: Applications to addictive behaviors. Am Psychol.

[48] Velicer W, Cumming G, Fava J, Rossi J, Prochaska J, Johnson J (2008). Theory testing using quantitative predictions of effect size. Appl Psychol.

[49] Halko S, Kientz J (2010). Personality and persuasive technology: An exploratory study on health-promoting mobile applications. Proceedings of the 5th International Conference on Persuasive Technology (PERSUASIVE 2010).

[50] Rubin J, Chisnell D (2008). Handbook of Usability Testing: How to Plan, Design, and Conduct Effective Tests. 2nd edition.

[51] Baretta D, Perski O, Steca P (2019). Exploring users' experiences of the uptake and adoption of physical activity apps: Longitudinal qualitative study. JMIR Mhealth Uhealth.

[52] Niedderer K, Mackrill J, Clune S, Lockton D, Ludden GD, Morris A, Cain R, Gardiner E, Gutteridge R, Evans M, Hekkert P (2014). Creating Sustainable Innovation Through Design for Behaviour Change: Full Report.

