# Supplementary material for: A Mobile App Adopting an Identity Focus to Promote Physical Activity (MoveDaily): Iterative Design Study
Source: JMIR Mhealth Uhealth. 2020 Jun 15;8(6):e16720. doi: 10.2196/16720 (PMC7325000; doi:10.2196/16720)
Supplement: Multimedia Appendix 1 [file mhealth_v8i6e16720_app1.pdf]

move daily

Welcome to a new day of MoveDaily!

Good to have you here.

Let's start! press **ENTER**

1 → Welcome back (...) !*

What was your name again?

OK ✓ press **ENTER**

0% completed

Powered by **Typeform**

1 → Welcome back (...) !*

What was your name again?

Floris

2 → We recently decided on a **daily** movement.
Did you do your movement today, Floris?

*

Y Yes

N No

50% completed

Powered by Typeform

Y Yes ✓

N No

3 → Awesome! Don't forget to reward yourself with a pat on the back and take a moment to appreciate how it feels.*

Now that you successfully performed your movement.

A very happy

B happy

C not so happy

D not happy

Other

E

“ Hold that feeling and use it as fuel! See, Floris is a person who moves more every day :-)

woah I'm so AWESOME press ENTER

100% completed

Powered by Typeform

A very happy

B happy

C not so happy

D not happy

Other

E

“ Hold that feeling and use it as fuel! See, Floris is a person who moves more every day :-)

woah I'm so AWESOME press **ENTER**

Powered by **Typeform**

Other

E

“ Hold that feeling and use it as fuel! See, Floris is a person who moves more every day :-)

woah I'm so AWESOME press ENTER

Submit press ENTER

100% completed

Powered by Typeform

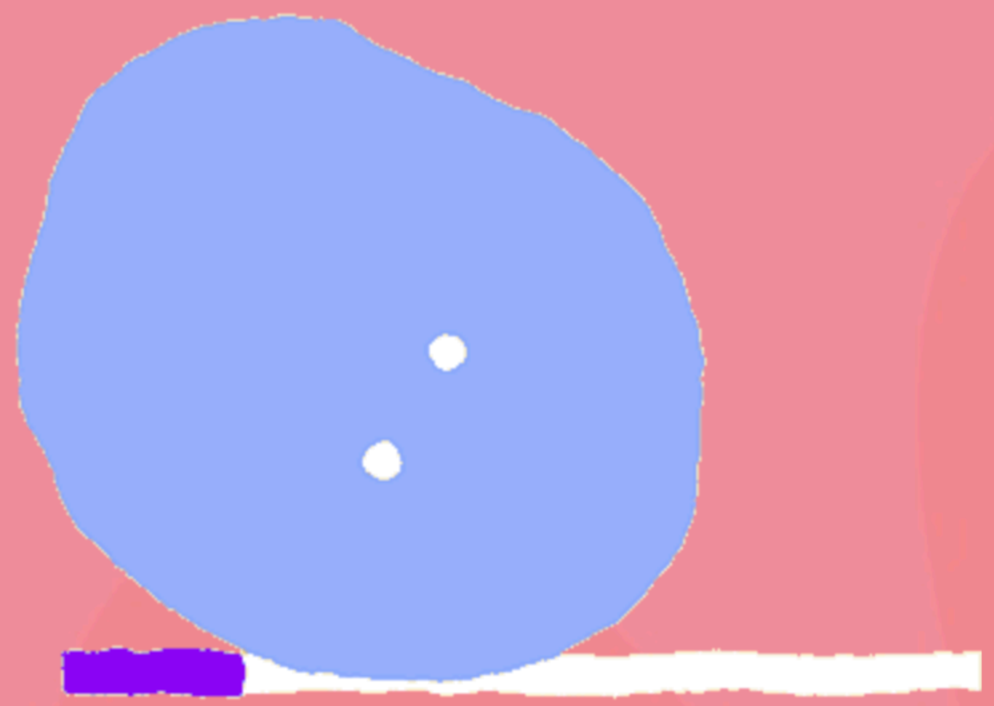

I will see you tomorrow Floris for the next day of MoveDaily!

Ask awesomely... Create a **typeform**
