# Supplementary figures and images for: A Mobile App Adopting an Identity Focus to Promote Physical Activity (MoveDaily): Iterative Design Study
Source: JMIR Mhealth Uhealth. 2020 Jun 15;8(6):e16720. doi: 10.2196/16720 (PMC7325000; doi:10.2196/16720)

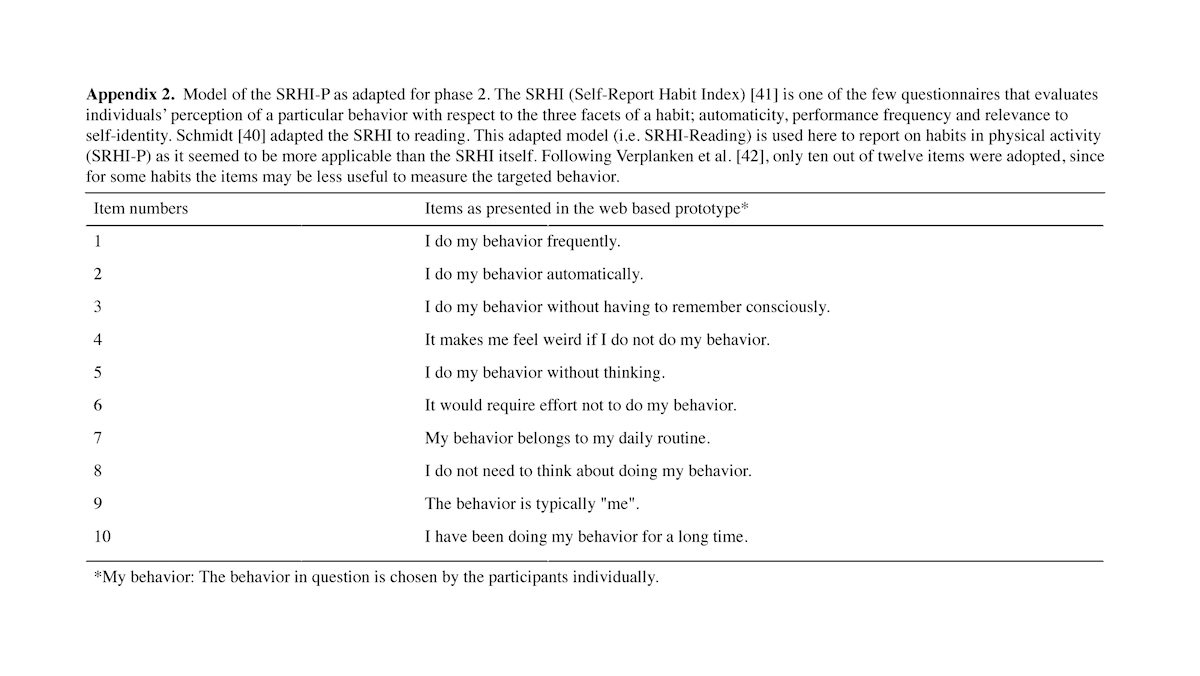

Supplement: Multimedia Appendix 2 [file mhealth_v8i6e16720_app2.png]
